# A new roadmap for the breeding of disease-resistant and high-yield crops

**DOI:** 10.1007/s44154-021-00023-0

**Published:** 2021-12-29

**Authors:** Yiming Wang, Suomeng Dong

**Affiliations:** 1 Key Laboratory of Biological Interaction and Crop Health, Department of Plant Pathology, Nanjing Agricultural University, Nanjing, 210095 China; 2 Key Laboratory of Integrated Management of Crop Disease and Pests, Ministry of Education, Nanjing Agricultural University, Nanjing, China

**Keywords:** Rice, Immunity, Ca^2+^ signaling, Reactive oxygen species; *ROD1*; *Pigm*

## Abstract

Breeding of disease-resistant and high-yield crops is essential to meet the increasing food demand of the global population. However, the breeding of such crops remains a significant challenge for scientists and breeders. Two recent discoveries may help to overcome this challenge: the discovery of a novel molecular framework to fine-tune disease resistance and yields that includes epigenetic regulation of antagonistic immune receptors, and the discovery of a Ca^2+^ sensor-mediated immune repression network that enables the transfer of subspecies-specific and broad-spectrum disease resistance. These breakthroughs provide a promising roadmap for the future breeding of disease resistant crops.

## Introduction

Global food production needs to increase by at least 60% to meet the food demand of the estimated global population of 10 billion people in 2050 (Food and Agriculture Organization, 2019). To meet this goal, a significant increase of crop production is needed. It is estimated that the global crop loss of 17–30%, depending on different crops, is due to plant pathogens and pests (Savary et al. 2019). However, improving crop resistance to pathogens and pests is challenging, since host resistance is frequently accompanied by growth disadvantage and yield penalty, which is known as the defense-growth tradeoff (Yang et al., 2012). To address this challenge, extensive research efforts have been aimed at generating pathogen-resistant crops with less yield penalty.

Zuhua He’s group, from the CAS Center for Excellence in Molecular Plant Sciences, study the mechanistic interaction between resistance and yield and aim to find a balanced solution for crop breeding. Gumei4, a highly resistant rice variety containing the *Pigm* resistance locus, has been used to improve the resistance of rice against the rice blast fungus for over 50 years. However, the molecular mechanism of *Pigm*-mediated resistance has remained unclear until recently. Deng et al. developed near-isogenic rice lines containing the *Pigm* resistance locus (NIL-Pigm) (Deng et al., 2006). By map-based cloning and gamma-ray-mutagenized Gumei4 populations, the *Pigm* locus was functionally identified; it contains three intact nucleotide-binding leucine-rich repeats (NLR) receptors, 10 potential pseudogenes, and one retrotransposon. The NLR receptor *Pigm Resistant* (*PigmR*) was further identified as a broad resistance gene against the rice blast fungus. Transgenic rice plants expressing *PigmR* significantly increased their resistance against the rice blast fungus but also showed yield penalty. However, Deng et al. also noticed that no significant yield penalty was observed in NIL of *Pigm* in either *indica* or *japonica* rice varieties, suggesting that a defense-growth balance was maintained by genes in the *Pigm* locus.

The ectopic expression of another NLR receptor in the *Pigm* locus, *Pigm Susceptible* (*PigmS*), reduces *PigmR*-mediated resistance by forming *PigmR-PigmS* heterodimers. This reduction of host immunity in *PigmS*-expressing plants is accompanied by an increase in grain yield due to an increase in seed settings. However, the mechanism of the *PigmR-PigmS*-mediated defense-growth tradeoff inside the host is still unclear. Deng et al. further noticed that the expression of *PigmS* under native promoters did not affect disease resistance mediated by *PigmR*, suggesting that the expression pattern may be essential for the function of *PigmS*.

RNA-directed DNA methylation (RdDM) is required for the epigenetic silence of plant transposable elements (Cui and Cao, 2014). The lower expression of *PigmS* in leaves was affected by two tandem miniature transposons (MITE1 and MITE2) in its promoter region (Deng et al., 2017). Accumulation of 24-nucleotide-small interfering RNA is responsible for the RdDM-mediated silencing of *PigmS* at MITE1 and MITE2 regions in leaves. Therefore, under non-disease conditions, the total yield balance was mediated by *PigmR*-decreased grain weight and *PigmS*-enhanced seed setting. These findings demonstrated that epigenetic regulation of the NLR-repressive pair gene balances the defense-growth tradeoff in rice.

NLR genes are highly polymorphic in rice germplasm and typically confer specific resistances. Is it possible to modulate conserved genes to enhance the broad resistance of plants against multiple pathogens? Recently, Zuhua He’s group identified a broad-spectrum resistant rice variant, *Resistance of rice to disease1* (*rod1*), through large-scale screening of germplasm and breeding collections. The *rod1* mutant exhibits high resistance against the rice blast fungus, rice bacterial blight, and rice sheath blight, which are the major threats to rice productivity (Gao et al. 2021). Map-based cloning showed that *ROD1* encodes a C2-domain Ca^2+^ sensor protein exhibiting Ca^2+^ binding activity. Loss of Ca^2+^ binding disables the lipid binding and plasma membrane localization of ROD1, causing failure to rescue growth retardation and immune activation in *rod1* (Gao et al. 2021). These findings illustrate that the Ca^2+^ binding activity is crucial for the function of ROD1.

Interestingly, the *rod1* mutant exhibits high accumulation and induction levels of reactive oxygen species (ROS) in rice, highlighting a potential link between Ca^2+^ and ROS signaling. The ROD1-interacting protein CatB, a ROS-scavenging enzyme, was detected through a yeast-two-hybrid screening system. These findings illustrate the possible role of ROD1 in ROS modulation through CatB. ROD1 recruits the localization of CatB from the peroxisome to the plasma membrane and promotes the ROS-scavenging activity of CatB (Gao et al. 2021). Moreover, co-expression of ROD1-CatB significantly reduces coiled-coil NLR-mediated cell death and ROS accumulation in tobacco leaves. These findings illustrate that the ROD1-CatB interaction is required to suppress rice immunity through modulating ROS accumulation. Consistent with the defense-growth tradeoff hypothesis, the *rod1* mutant exhibits a phenotype of compromised plant growth and reduced productivity. This raises the question of whether it is possible to enhance pathogen resistance without yield penalty. To answer this question, Gao et al. identified the natural variations of ROD1 from 262 cultivated Asian rice accessions. Interestingly, they found a non-synonymous nucleotide polymorphism, SNP1^A/C^ (an amino acid change from proline to threonine), which contributes to subspecies-specific disease resistance. The ROD1 SNP1^A^ is mainly retained in *indica* rice varieties, whereas SNP1^C^ is predominant in both wild rice and *japonica* rice varieties. A complementation assay using ROD1 SNP1^A^ and SNP1^C^ was carried out in the *rod1* mutant and showed that ROD1 SNP1^C^ affected neither pathogen resistance nor plant growth. However, ROD1 SNP1^A^, which showed a reduction of H_2_O_2_ hydrolysis activity, transferred the enhanced disease resistance without growth repression. This result is consistent with evolutionary analysis showing that the ROD1 in *indica* rice undergoes differential selection during rice adaptation.

The identification of *PigmR* and *ROD1*, which either confers broad-spectrum blast resistance or suppresses immunity, was part of a large-scale study screening highly resistant plants in the field. *PigmR* exhibits genus-specific resistance against the rice blast fungus, whereas *rod1* exhibits broad resistance against multiple pathogens. Although these two genes provide different molecular mechanisms, their good performances in terms of pathogen resistance under natural conditions make them promising candidates for utilizing those resistances in future breeding processes. The maize *ZmROD1* mutant also exhibits enhanced resistance against the pathogenic fungus *R. solani*, suggesting that ROD1 is functionally conserved across cereal plants (Gao et al. 2021). Moreover, pathogen-derived selection pressure during long-term co-evolution resulted in the maintaining of beneficial gene variations in plants. A single SNP in ROD1 does not cause differences in growth but does improve field disease resistance. These findings provide a roadmap for the breeding of highly resistant crops with less yield penalty. Utilizing the available information on natural variation may provide a deeper understanding of molecular and evolutionary mechanisms in plant immunity.

Information on the crosstalk between different signaling pathways in plants remains incomplete. Ca^2+^ and ROS are required for multiple processes in plants, including development and biotic and abiotic responses. ROD1 bridges Ca^2+^ signaling to ROS signaling in rice defenses, and this mechanism is likely to occur in other plants. A better understanding of signaling crosstalk, especially the identification of joint points of signaling networks, can lead to a better understanding of tradeoff between defense and growth, biotic and abiotic responses, and other mechanisms. Identifying these antagonistic biological processes provides a promising outlook for the future breeding of resistant crops.

## Data Availability

Not applicable.

## Abbreviations

CatB: Catalase B
NIL: Near-isogenic rice lines
NLR: nucleotide-binding leucine-rich repeats
PigmR: Pigm Resistant
PigmS: Pigm Susceptible
RdDM: RNA-directed DNA methylation
ROD1: RESISTANCE OF RICE TO DISEASE1
ROS: Reactive oxygen species
SNP: Synonymous nucleotide polymorphism
