# Correction to: A new roadmap for the breeding of disease-resistant and high-yield crops

**DOI:** 10.1007/s44154-022-00037-2

**Published:** 2022-02-11

**Authors:** Yiming Wang, Suomeng Dong

**Affiliations:** 1 Key Laboratory of Biological Interaction and Crop Health, Department of Plant Pathology, Nanjing Agricultural University, Nanjing, 210095 China; 2 Key Laboratory of Integrated Management of Crop Disease and Pests, Ministry of Education, Nanjing Agricultural University, Nanjing, China

**Correction to: Stress Biol 1, 21 (2021)**

**https://doi.org/10.1007/s44154-021-00023-0**

Following publication of this article (Wang & Dong, 2021), it is reported that this article contained two errors.

1. The name of the 2nd author should be ‘Suomeng Dong’ and this has been reflected in this Correction;
2. The reference ‘Hout B et al., 2014’ and its corresponding citation should be removed.

The original article has been updated.
